# A Rare Finding Altering the Surgical Approach of Laparoscopic Cholecystectomy: A Case Report

**DOI:** 10.7759/cureus.77957

**Published:** 2025-01-25

**Authors:** Mooza Alabbasi, Thamer AlAbbasi

**Affiliations:** 1 General Surgery, Bahrain Defence Force Hospital, Riffa, BHR

**Keywords:** congenital anomalies of the liver, ectopic hepatic tissue, gallbladder removal, laparoscpic surgery, minimally invasive laparoscopy

## Abstract

Ectopic hepatic tissue occurs due to an uncommon failure in embryological liver development, a phenomenon rarely described in the literature. We report a case of a 45-year-old male who presented to the emergency department with abdominal pain. The patient was investigated radiologically, and ectopic hepatic tissue and calcular cholecystitis were diagnosed. As a result, the patient underwent laparoscopic cholecystectomy. The patient recovered well postoperatively, had no complications, and was discharged the day after the operation. It is imperative to be attentive to ectopic hepatic tissue, including its complications and long-term prognosis.

## Introduction

Congenital liver anomalies are rare and occur as a result of a failure of embryological development of the liver. It is rarely illustrated in surgical literature yet has been reported in a few case reports. Internationally, its incidence varies between 0.24% to 0.47% [1]. It is a rare encounter involving hepatic tissue in various sites, such as the gallbladder, omentum, thorax, and retroperitoneum [2]. As a result of this asymptomatic entity, most cases are diagnosed either perioperatively, in the autopsy, or preoperatively with computed tomography if a high index of clinical suspicion arises. More recently, some cases of ectopic liver reported an incidence of malignant transformation to hepatocellular carcinoma (HCC) [3]. We report a case of a 45-year-old male who presented with biliary colic and was diagnosed with calculous cholecystitis and ectopic liver tissue preoperatively that altered the surgical approach of his management.

## Case presentation

**Figure 1:**
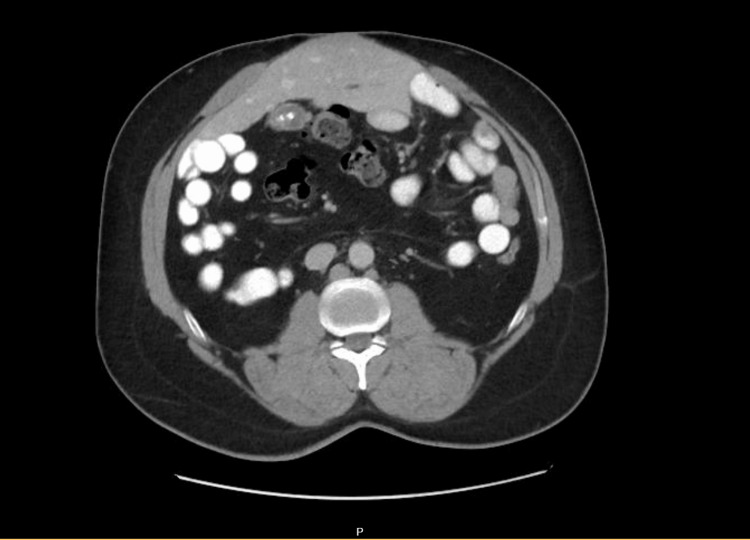
Axial section of the abdomen showing ectopic left lobe of the liver near umbilicus.

A 45-year-old male presented to the emergency department with symptoms suggestive of intestinal obstruction with abdominal pain aggravated by ingesting fatty food associated with abdominal distention. There was no associated nausea or vomiting and the patient maintained regular bowel habits. The patient was hemodynamically stable and, on examination of his abdomen, showed a soft abdomen with epigastric and paraumbilical tenderness. A decision to proceed with a CT abdomen and pelvis has been made to rule out intestinal obstruction. This imaging was carried out by the emergency department physician before being referred to the surgical department. The CT has reported a soft tissue density noted over the anterior to the bowel with a small connection with the liver showing internal vascularity with flow derived from the portal vein, likely the ectopic left lobe of the liver. The bowel demonstrated no evidence of obstruction (Figure 1). The characteristics of the lesion on CT scanning demonstrated an irregular mass with a homogenous density similar to the density of hepatic tissue.

**Figure 2:**
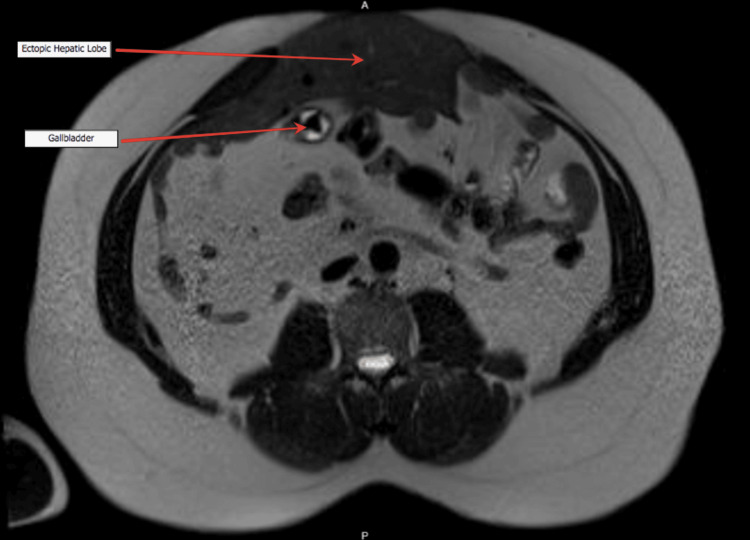
Magnetic resonance cholangiopancreatography showing the thin-walled, distended gallbladder with multiple small stones inside

**Figure 3:**
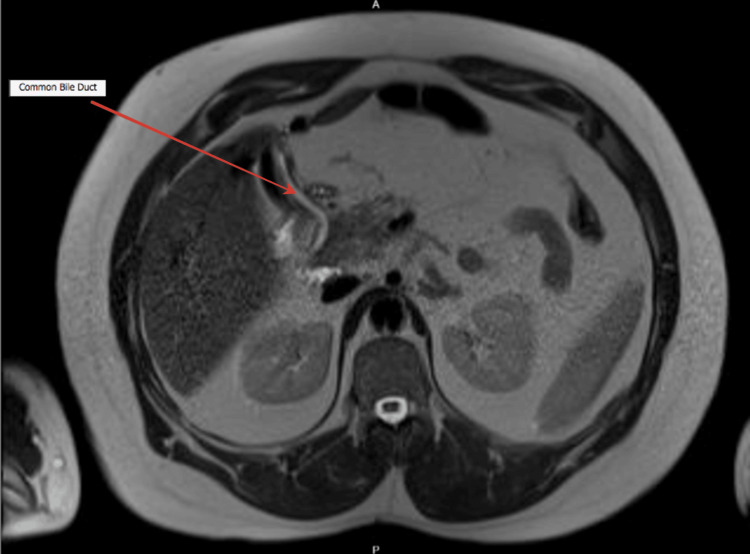
The common bile duct measures 4.8mm at its widest diameter with no ductal stones on magnetic resonance cholangiopancreatography

Later, the patient presented to the surgical outpatient clinic, and a decision was made to proceed with magnetic resonance cholangiopancreatography (MRCP) secondary to abnormal anatomy with possible cholecystitis. MRCP is a useful radiological tool and the most effective preoperative examination method as it delineates the anatomy. Using a T2-weighted sequence, the MRCP indicated calcular gallbladder disease (Figures 2-3). Hence, the patient was booked electively for laparoscopic cholecystectomy.

Laboratory investigations were unremarkable, with normal liver function tests. The patient underwent laparoscopic cholecystectomy with three port incisions in the lateral left paraumbilical and lateral supra- and infra-paraumbilical regions. The camera was inserted in the left lateral paraumbilical incision, and two more ports were inserted under direct vision. Adhesiolysis was performed due to extensive small bowel adhesions to the anterior abdominal wall and liver. The gallbladder was identified, adherent to the anterior abdominal wall, with multiple gallstones resected, extracted, and sent for histopathology. A drain was inserted, and the patient was kept in the hospital for two days to observe and monitor hemodynamics. Later, the patient was discharged with an uneventful recovery and was advised for outpatient follow-up in the surgical clinic after two weeks.

**Figure 4:**
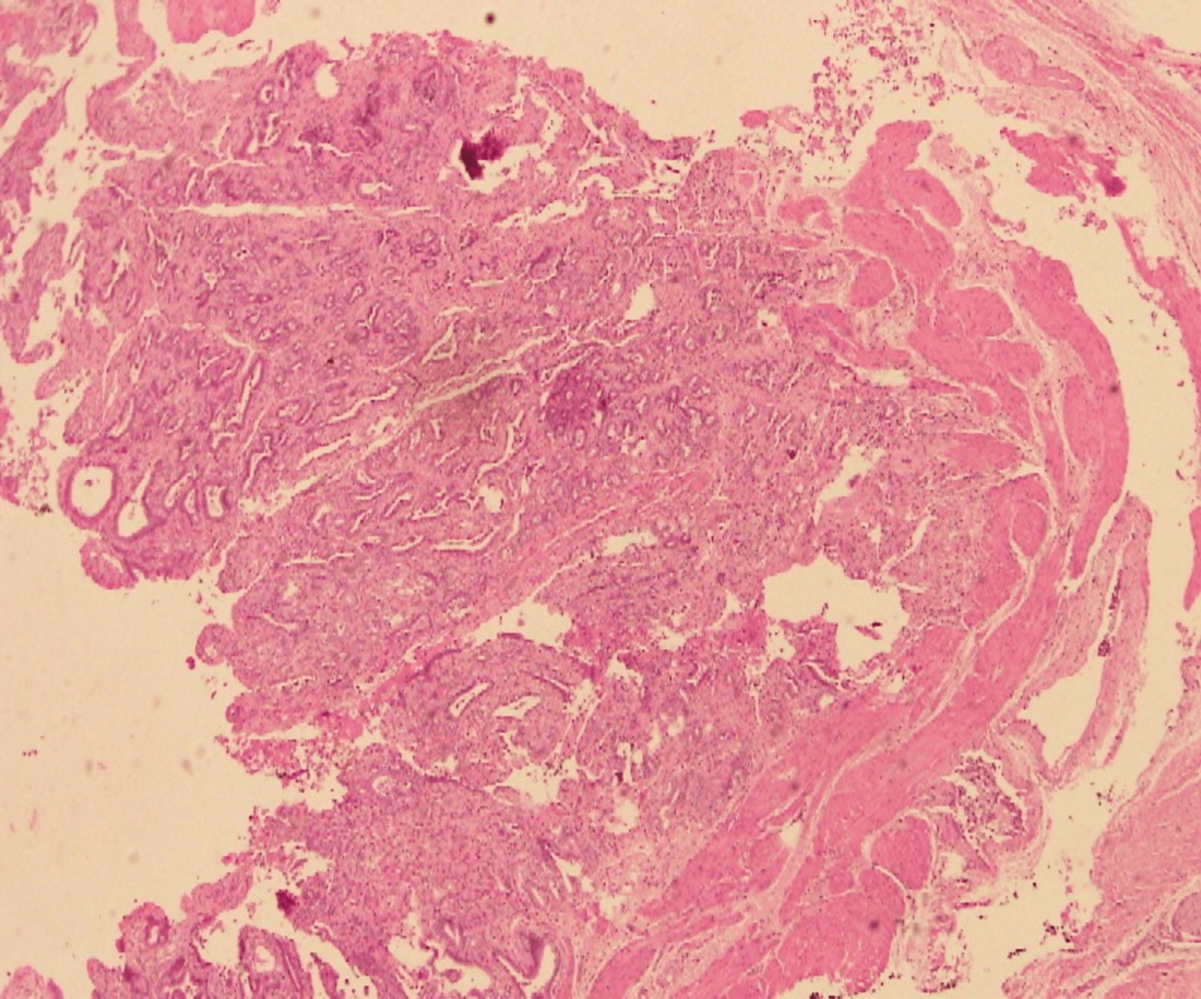
Adenomyomatous hyperplasia with reactive surface atypia

Histopathology confirmed acute-on-chronic calculous cholecystitis with adenomyomatous hyperplasia, accompanied by reactive surface atypia (Figure 4).

## Discussion

Ectopic hepatic tissue is an infrequent finding in clinical practice. There are four main types of ectopic hepatic tissue described in surgical literature: ectopic liver; microscopic ectopic liver found in the wall of the gallbladder; an accessory liver lobe adherent to the mother liver by a stalk, and a small accessory liver lobe adherent to the mother liver [4]. The incidence of ectopic hepatic tissue is challenging to estimate as the majority of the cases are asymptomatic and are identified incidentally either during laparotomy, laparoscopy or during an autopsy [5].

It has been postulated that an ectopic liver arises at numerous locations, either the development of an accessory lobe with regression or atrophy of the original junction to the mother liver, displacement of a specific portion of the pars hepatica to different areas, or entrapment of a nest of cells in the site of the foregut following the closure of the umbilical ring or diaphragm [5].

Having ectopic liver tissue leads to certain complications such as torsion, fatty changes with the transition to cirrhosis, and malignant degeneration to hepatocellular carcinoma [1]. Ectopic hepatic tissue typically has a standard histological outline with regular lobules, a central vein, and typical portal structures. It has an increased risk of hepatocellular carcinoma; the increased risk of this malignant transformation remains unclear. However, it has been postulated that there is insufficient biliary drainage or reduced blood supply to the ectopic hepatic tissue [6].

In our case, the patient initially presented to the emergency with symptoms suggestive of intestinal obstruction. A decision to proceed with a CT abdomen has been made to rule this differential out. As a result, ectopic hepatic tissue has been identified along with calcular cholecystitis. Hence, the reported findings on imaging changed the traditional way of proceeding with laparoscopic cholecystectomy. In essence, ectopic liver tissue remains asymptomatic and is occasionally discovered pre-operatively with imaging or during laparoscopy [7], as the case with our patient in the present case report. If the patient is symptomatic, the principal complaint is typically upper abdominal pain due to the above-mentioned complications.

Detection of ectopic hepatic tissue before operation with the imaging guide is rare. The diagnosis of ectopic hepatic tissue should be considered when a mass is being identified on the gallbladder wall. Biopsies of the ectopic hepatic tissue should be deferred due to the risk of hemorrhage and the possibility of malignant transformation to hepatocellular carcinoma [8].

In our present case, the patient was planned for laparoscopic cholecystectomy. The procedure was done uneventfully, and a different approach has been decided to remove the gallbladder. Thus, advances in surgical techniques allow preoperative recognition of the anatomy [9]. Multiple techniques have been described, while no specific method has been proven superior, and therefore our approach was decided based on careful preoperative planning.

## Conclusions

Ectopic liver is an uncommon entity reported in the surgical literature. Identifying hepatic tissue is crucial as it increases the operative time and the need for follow-up to rule out any possible complications. In addition, the need for histopathological examination of the resected gallbladder as it carries a risk of malignant degeneration.
